# Antiamoebic activity of plant-based natural products and their conjugated silver nanoparticles against *Acanthamoeba castellanii* (ATCC 50492)

**DOI:** 10.1186/s13568-020-0960-9

**Published:** 2020-02-03

**Authors:** Areeba Anwar, Elaine Lim Siew Ting, Ayaz Anwar, Noor ul Ain, Shaheen Faizi, Muhammad Raza Shah, Naveed Ahmed Khan, Ruqaiyyah Siddiqui

**Affiliations:** 1 Department of Biological Sciences, School of Science and Technology, Sunway University, Subang Jaya, 47500, Selangor, Malaysia; 2 Department of Chemistry, University of Karachi, Karachi, 75270 Pakistan; 3 HEJ Research Institute of Chemistry, International Center for Chemical and Biological Sciences, University of Karachi, Karachi, 75270 Pakistan; 4 Department of Biology, Chemistry and Environmental Sciences, American University of Sharjah, Sharjah, 26666 United Arab Emirates

**Keywords:** Quercetin, *Polyalthia longifolia*, *Caesalpinia pulcherrima*, Silver nanoparticles, Antiamoebic, *Acanthamoeba*

## Abstract

*Acanthamoeba* spp. are the causative agent of *Acanthamoeba* keratitis and granulomatous amoebic encephalitis (GAE). The current options to treat *Acanthamoeba* infections have limited success. Silver nanoparticles show antimicrobial effects and enhance the efficacy of their payload at the specific biological targets. Natural folk plants have been widely used for treating diseases as the phytochemicals from several plants have been shown to exhibit amoebicidal effects. Herein, we used natural products of plant or commercial sources including quercetin (QT), kolavenic acid (PGEA) isolated from plant extracts of *Polyalthia longifolia var pendula* and crude plant methanolic extract of *Caesalpinia pulcherrima* (CPFLM) as antiacanthamoebic agents. Furthermore, these plant-based materials were conjugated with silver nanoparticles (AgNPs) to determine the effects of the natural compounds and their nanoconjugates against a clinical isolate of *A. castellanii* from a keratitis patient (ATCC 50492) belonging to the T4 genotype. The compounds were conjugated with AgNPs and characterized by using ultraviolet visible spectrophotometry and atomic force microscopy. Quercetin coated silver nanoparticles (QT-AgNPs) showed characteristic surface plasmon resonance band at 443 nm and the average size distribution was found to be around 45 nm. The natural compounds alone and their nanoconjugates were tested for the viability of amoebae, encystation and excystation activity against *A. castellanii.* The natural compounds showed significant growth inhibition of *A. castellanii* while QT-AgNPs specifically exhibited enhanced antiamoebic effects as well as interrupted the encystation and excystation activity of the amoebae. Interestingly, these compounds and nanoconjugates did not exhibit in vitro cytotoxic effects against human cells. Plant-based compounds and extracts could be an interesting strategy in development of alternative therapeutics against *Acanthamoeba* infections.

## Introduction

*Acanthamoeba* is ubiquitously found in nature (Martinez and Visvesvara 1997). The life cycle of *Acanthamoeba* consists of an active stage called trophozoites, and a dormant cyst stage (Marciano-Cabral and Cabral 2003). *Acanthamoeba* converts to cysts when the environment is not optimal for survival (Khan 2006). Cysts are very resistant in order to survive under harsh conditions. In cysts stage, *Acanthamoeba* is resistant to chlorine, biocides and antibiotics (De Jonckheere and Van de Voorde 1976; Lloyd et al. 2001; Turner et al. 2000). The cysts may survive under low temperature ranging from 0 to 2 °C (Brown and Cursons 1977).

*Acanthamoeba* species have been characterized by using the rRNA sequence and are separated into 21 genotypes (Fuerst et al. 2015). The T4 genotype of *Acanthamoeba* spp. is found to be most associated with infections (Niyyati et al. 2016). *Acanthamoeba* T4 can cause *Acanthamoeba* keratitis (AK) and granulomatous amoebic encephalitis (GAE) (Khan 2006; Visvesvara et al. 2007). Contact lens wearers (CLW) were proven to have a higher risk of contracting AK due to improper cleaning and extended wearing of contact lenses (CL). There are estimated 45million people that are using CL daily in the United States in 2016 and CL wearers account for 85% of the AK cases (Cope et al. 2017; Niyyati et al. 2016). On the other hand, GAE is a fatal disease as the treatment remains difficult because of the limited penetration of drugs through the blood brain barrier (BBB) as it is a highly selective barrier when compared to peripheral endothelium (Miller 1999). Due to the difficulties in treatment with current therapeutic options, development of antiacanthamoebic agents is urgently needed.

Natural folk plants are widely researched for treating various diseases. The phytochemicals extracted from a variety of plants exhibit amoebicidal effects. For example,* Allium sativum*,* Origanum syriacum*,* Origanum laevigatum*,* Arachis hypogaea*,* Curcuma longa* L.,* Pancratium maritimum* L.,* Pterocaulon polystarchyum*,* Croton isabelli*,* Croton pallidulus*,* Croton ericoides* etc. have shown antiamoebic effects against both trophozoite and cyst stages of amoebae (Polat et al. 2008; Degerli et al. 2012; El-Sayed et al. 2012; Vunda et al. 2012). Therefore, research on plants extract to identify their amoebicidal effect is a promising avenue.

**Fig. 1:**
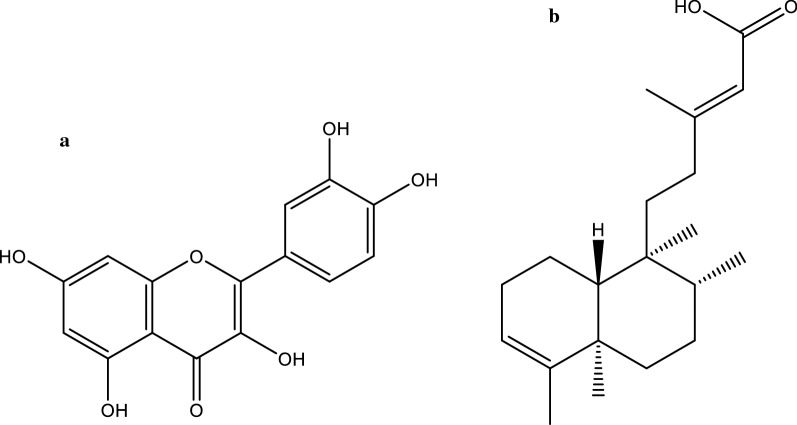
Chemical structure of quercetin **a** and **b** Kolavenic acid (PGEA)

Quercetin, 3,5,7,3′,4′-Pentahydroxyflavone (Fig. 1a) is a light-yellowish bioactive flavonoid compound isolated from an oak named *Querces oak*. Quercetin is ubiquitously found in fruits and vegetables (Brown et al. 2019). This bioactive compound has been studied on cells and animal models for antioxidative, antibacterial, antiviral, anti-inflammatory and anticancer features (David et al. 2016). In a study conducted by Amin et al. (2016), quercetin expressed the highest antimicrobial activity against *Staphylococcus aureus*. Moreover, in a recent study, *Artemisia argyi* leaves which were found to contain quercetin exhibited antiamoebic features and cytotoxicity against *A. castellanii* (Kolören et al. 2019).

Kolavenic acid (PGEA) (Fig. 1b) is a diterpene (16-oxo-cleroda-3,13(14)-E-diene-15-oic acid) that can easily be isolated from widely occurring plant *Polyalthia longifolia* from the family *Annonaceae*. This plant is known in south Asia as ‘Ulta Ashok’. Extracted compounds from *Polyalthia longifolia* have expressed several biological functions proven through therapeutic experiments (Katkar et al. 2010). Previously, Rashid et al. (1996) showed PGEA to cause inhibition of bacteria and several fungal strains that were resistant to antibiotics. Another plant, *Caesalpinia pulcherrima* has been used in treating liver infections, intermittent fever, skin diseases, diarrhea, inflammation etc. (De Padua et al. 1978; Patel et al. 2010). The flower of *C. pulcherrima* are famous folklore herbal medicine for erysipelas and optical inflammation (De Padua et al. 1978). Research has shown that *C. pulcherrima* expressed antifungal activity against *Candida albicans* and *Cryptococcus neoformans* (Vivek et al. 2013). The methanolic extract of *C. pulcherrima* has shown an inhibition zone of 1.3 cm and 1.2 cm for *C. albicans* and *C. neoformans* (Vivek et al. 2013).

Silver nanoparticles (AgNPs) have been shown to possess antimicrobial effects against microorganisms such as yeast, fungi, bacteria and parasites (Kim et al. 2007). Green-synthesized silver nanoparticles have tremendous potential in antimicrobial chemotherapy (Ahmed et al. 2016). Plant extracts and purified natural products conjugated with nanoparticles have high therapeutic values for drug development against infectious diseases (Kuppusamy et al. 2016). Recently, various plant extracts and purified natural products coated nanoparticles have shown potential translational values against *A. castellanii*. For example, *Aloe vera* conjugated with silver nanoparticles, *Pongamia pinnata* conjugated with zinc oxide (Dinesh et al. 2015; Sundrarajan et al. 2015).

Hence, based on above literature, this study was designed to test the antiacanthamoebic effects of plant-based compounds and extracts including quercetin, PGEA, and CPFLM for the first time. Moreover, these natural products were conjugated with silver nanoparticles to assess the effect of organic v/s nanomaterials against *A. castellanii*.

## Materials and methods

### Materials

The chemicals including Trypan blue, phosphate-buffered saline (PBS), non-nutrient agar, proteose peptone, yeast extract, d-glucose, Roswell Park Memorial Institute 1640 medium (RPMI-1640), sodium dodecyl sulfate (SDS), silver nitrate (AgNO_3_), methanol and dimethylsulfoxide (DMSO) were purchased from Thermo Fisher Scientific (Massachusetts, USA). Chlorhexidine was purchased from Sigma-Aldrich (San Francisco, USA). Quercetin was purchased from Sigma-Aldrich (San Francisco, USA), while PGEA isolation and preparation of CPFLM extracts were carried out as reported previously (Khan et al. 2017).

### *Acanthamoeba castellanii* culture

A clinical isolate of *A. castellanii* from a keratitis patient (ATCC 50492) was cultured with PYG medium containing proteose peptone 0.75% (w/v), yeast extract 0.75% (w/v) and glucose 1.5% (w/v) at 30 °C in 75 cm^2^ tissue culture flask. The flasks were checked with the condition of the cells and the turbidity of the media to make sure the culture is free from bacterial contaminations. The media in the flask was changed after every 24 h of incubation. Moreover, when the cell confluency is 70% and above, sub-culturing was performed.

### Silver nanoparticles synthesis

The synthesis of natural products coated AgNPs was done by adding silver nitrate (AgNO_3_) aqueous solution with a reducing agent sodium borohydride in the presence of QT, PGEA and CPFLM. 500 μL QT and PGEA (0.1 mM) were mixed with 500 μL 0.1 mM AgNO_3_ in the microcentrifuge tubes, followed by addition of 20 μL of sodium borohydride (5 mM) and sonicated for 1 h. The colour of reaction changed to yellow–brown upon addition of reducing agent. CPFLM is methanolic extract of *Caesalpinia pulcherrima* flower. 30 mg of dried extract was dissolved in 30 mL of methanol and made up to 50 mL volume with deionized water. The solution was then filtered and 10 mL of the filtrate was added in 150 mL of silver nitrate solution (1 mM). The solution was stirred at 25 °C temperature for 3 h. Nanoparticle formation was evidenced with the visual color change of the mixture solution from colorless to yellow. These colloidal suspensions were subjected to characterization for the confirmation of nanoparticles synthesis.

### Characterization of silver nanoparticles

The conjugated AgNPs were characterized by using Ultraviolet–Visible Spectrophotometer (UV–Vis) (Thermo Fischer, Evolution 210) and atomic force microscopy (AFM). The solvents for each drug were used as the blank and obtained a wavelength graph after running the test. For AFM, samples were loaded on mica surface and air dried, finally these samples were imaged on AFM instrument (Agilent, 5500) in tapping mode.

### Amoebicidal assay for *Acanthamoeba castellanii*

5 × 10^5^*A. castellanii* were incubated with 5 and 10 µM concentrations of natural products and their nanoconjugates with RPMI-1640 in each well (Sissons et al. 2006). Chlorhexidine was used as a positive control while RPMI-1640 alone as a negative control. Well plates were incubated for 24 h at 30 °C. The viability of *A. castellanii* was determined by adding 0.1% Trypan blue and counting live (unstained) amoeba.

### Encystation assay for *Acanthamoeba castellanii*

To assess the effects of these compounds on the inhibition of cyst formation, 5 × 10^5^*A. castellanii* trophozoites were incubated with natural products and their nanoconjugates at 5 and 10 µM concentrations with encystation media (10% glucose and 5 µM MgCl_2_) (Anwar et al. 2018b). Chlorhexidine was used as a positive control while encystation media as a negative control. Plates were incubated for 72 h at 30 °C. The formation of *A. castellanii* cysts was determined by adding 0.05% SDS which dissolves all trophozoites and live amoeba cysts were counted using haemocytometer.

### Excystation assay for *Acanthamoeba castellanii*

The cysts were prepared by inoculating 1 mL of cell suspension (cell pellet dissolved in PBS) onto non-nutrient agar plate and incubated at 30 °C for at least 14 days. The cysts were removed from the plates by adding PBS and scraping by using a cell scraper and were transferred into a 50 mL conical tubes. These tubes were centrifuged at 3000×*g* for 10 min, and the cell density was estimated by haemocytometer. 1 × 10^5^*A. castellanii* cysts were incubated with 5 and 10 μM concentrations of natural products and their nanoconjugates in PYG for 72 h at 30 °C (Anwar et al. 2018b). Trophozoites were counted by adding 0.1% Trypan blue for the cell viability.

### HaCaT keratinocyte cells culture

Human keratinocyte skin cells (HaCaT) (CVCL-0038, CLS, 300493) were purchased from Cell Line Service (CLS), Germany. HaCaT cells were routinely cultured in Roswell Park Memorial Institute (RPMI)-1640 supplemented with 10% of each foetal bovine serum and Nu-serum, 2 mM glutamine, 1 mM pyruvate, penicillin, streptomycin (100 units/mL and 100 μg/mL, respectively), vitamins and non-essential amino acids in 75-cm^2^ culture flasks as reported previously (Anwar et al. 2019a). After formation of confluent, uniform mono-layer (24 h), old media was aspirated, and cells were detached by using 2 mL trypsin. The cell suspension was centrifuged at 2500×*g* for 5 min. The obtained pellet was then re-suspended in 25 mL fresh cell growth media, and each well of a 96-well plate was seeded with 200 μL of this cell suspension and the plates were incubated in a 5% CO_2_ incubator with 95% humidity (at 37 °C for 24 h) until the uniform monolayer of cells was observed under light microscope.

### Cell cytotoxicity assays

Cytotoxicity of QT, PGEA, CPFLM, and their nanoconjugates towards human keratinocytes cells was tested by treating 5 and 10 µM with HaCaT monolayers in 96-well plates by using Lactate dehydrogenase (LDH) assay as described previously (Anwar et al. 2019a). The cells were incubated in a 37 *°*C, 5% CO_2_ incubator for 24 h. Untreated cells in RPMI-1640 only were taken as negative controls and 1% Triton X-100 was used as a positive control. Following the 24 h incubation, cell death was determined as extent of LDH release from damaged cells detected by LDH detection kit (Invitrogen) at 490 nm. % Cytotoxicity was calculated as follows:

$$\left( {{\text{Sample absorbance}} - {\text{negative control absorbance}}} \right)/({\text{Positive control absorbance}} - {\text{negative controlabsorbance}})\, \times \, 100.$$

### Statistical analysis

The significant differences were determined by using two sample T-test; two-tailed distribution comparing the average of two independent groups on an Excel sheet. A critical value of P < 0.05 is used for all the evaluations. In the graphical presentation, y-axis error bars are indicative of the mean ± standard errors of three experiments performed in duplicates.

## Results

### Natural compounds-conjugated AgNPs characterization

**Fig. 2:**
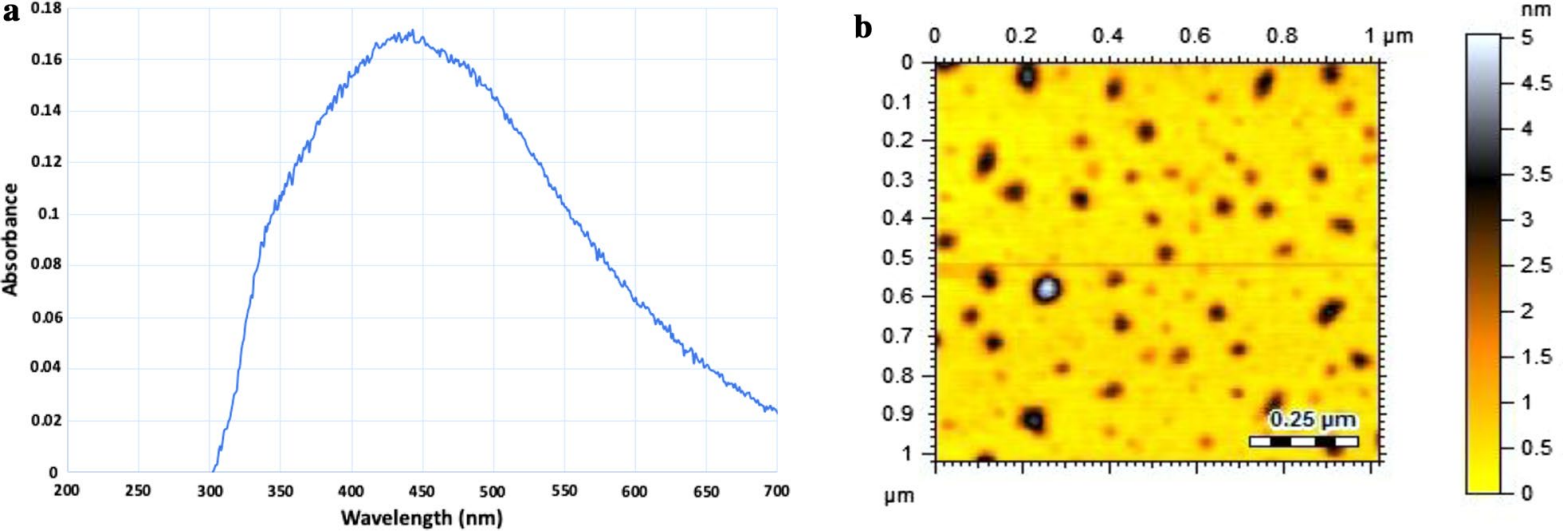
**a** UV–Vis spectra of quercetin conjugated silver nanoparticles (QT-AgNPs) showing characteristic surface plasmon resonance band at 443 nm confirming the formation of QT-AgNPs. **b** AFM topographic image recorded on an AFM instrument

The natural compounds-conjugated with AgNPs were characterized by UV–Vis spectrophotometry for the confirmation of AgNPs. Quercetin-AgNPs (QT-AgNPs) gave a characteristic surface plasmon resonance band (maximum absorption) at 443 nm as a representative example of stable silver nanoparticles of medium size (Fig. 2a) as compared to pure quercetin having a maximum absorption at 370 nm (He et al. 2012). AFM analysis was performed with QT-AgNPs as a representative example for the size and morphology of the AgNPs conjugated with natural compounds. The nanoconjugates were found to be spherical in shape and have an average diameter of 45 nm (Fig. 2b).

### QT, PGEA and CPFLM exhibited significant amoebicidal effects while silver nanoparticles conjugation enhanced their antiamoebic activity

**Fig. 3:**
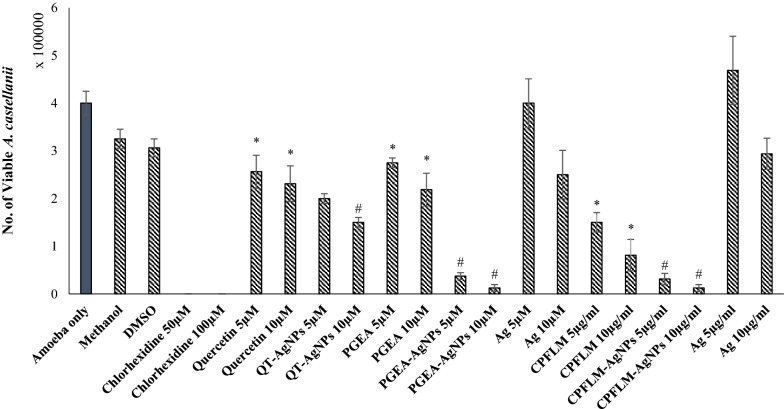
Amoebicidal effects of QT, PGEA, CPFLM and the nanoconjugates of the natural compounds above mentioned against *A. castellanii*. 5 × 10^5^*A. castellanii* was incubated with 5 and 10 μM (or mg/mL) of the natural compounds and the nanoconjugates for 24 h at 30 *°*C then the viability of *A. castellanii* was determined by staining with 0.1% Trypan Blue. The natural products QT, PGEA and CPFLM showed significant inhibition of *A. castellanii* growth when compared to the negative control (amoeba alone) (*P < 0.05, using two sample T-test, two-tailed distribution). Nanoparticles conjugation enhanced the antimaoebic activity of natural products as compared to AgNPs and compounds alone (*P < 0.05, using two sample T-test, two-tailed distribution). #shows significance as compared to compound and AgNPs alone

**Fig. 4:**
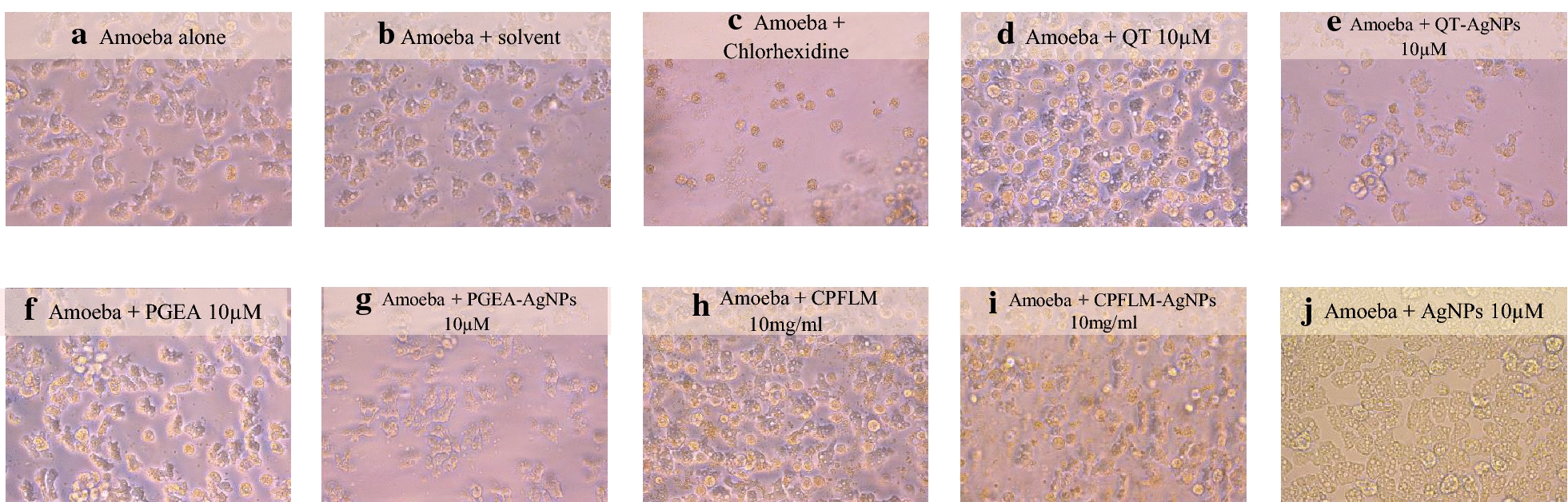
Representative effects of naturals compounds and nanoconjugates on inhibiting the viability of *A. castellanii*. **a** Amoeba alone; **b** amoeba + solvent control; **c** amoeba + chlorhexidine 100 μM (positive control); **d** amoeba + QT 10 μM; **e** amoeba + QT-AgNPs 10 μM; **f** amoeba + PGEA 10 μM; **g** amoeba + PGEA-AgNPs 10 μM; **h** amoeba + CPFLM 10 mg/mL; **i** amoeba + CPFLM-AgNPs 10 mg/mL; **j** amoeba + AgNPs alone 10 μM

Amoebicidal assay was performed to observe the effects of the natural products and their nanoconjugates on the viability of *A. castellanii*. 10 µM QT, PGEA and CPFLM significantly reduced the number of *A. castellanii* from 5 × 10^5^ to 2.3 × 10^5^, 2.18 × 10^5^ and 8.12 × 10^4^ respectively (Fig. 3). Furthermore, the conjugates showed significant reduction in amoebae viability as compared to natural products and AgNPs alone. Figure 4 shows representative images of the wells after *A. castellanii* treatment.

### QT-AgNPs showed enhanced effects against encystation of *A*. *castellanii*

**Fig. 5:**
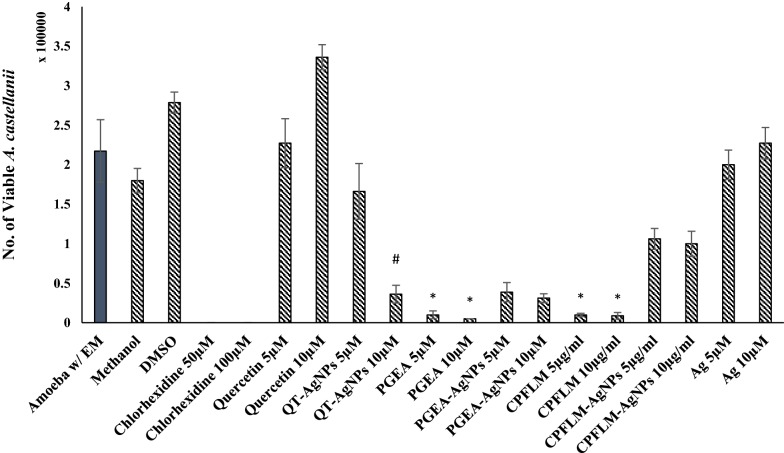
Effects of QT, PGEA, CPFLM and the nanoconjugates of the natural compounds above mentioned on the encystation of *A. castellanii*. 5 × 10^5^ amoebae were incubated with encystation media (E.M, including 10% glucose and 5 μM MgCl_2_) and respective 5 and 10 μM of compounds or nanoconjugates for 72 h at 30 *°*C. Then the cysts were counted after adding 0.05% of Sodium dodecyl sulfate (SDS). 10 μM of QT-AgNPs showed significantly enhanced encystation activity as compared to QT and AgNPs alone (#P < 0.05, using two sample T-test, two-tailed distribution). PGEA and CPFLM alone showed significant inhibition of *A. castellanii* encystation when compared to the negative control (amoeba + E.M) (*P < 0.05, using two sample T-test, two-tailed distribution)

QT alone did not show tendency to inhibit the encystation of *A. castellanii*, however, QT-AgNPs significantly inhibited the encystment. In case of PGEA and CPFLM, natural products showed significant inhibition of encystation by reducing the number of *A. castellanii* cysts to 5 × 10^3^ and 8.75 × 10^3^ when compared to the encystation medium alone which was considered as negative control (Fig. 5). As for nanoconjugates, PGEA-AgNPs and CPFLM-AgNPs also significantly reduced the number of cysts to 3.125 × 10^4^ and 1 × 10^5^ respectively as compared to negative control, but when compared to the natural products alone the PGEA-AgNPs and CPFLM-AgNPs did not show statistical significance.

### QT-AgNPs significantly inhibited the excystation of *A*. *castellanii*

**Fig. 6:**
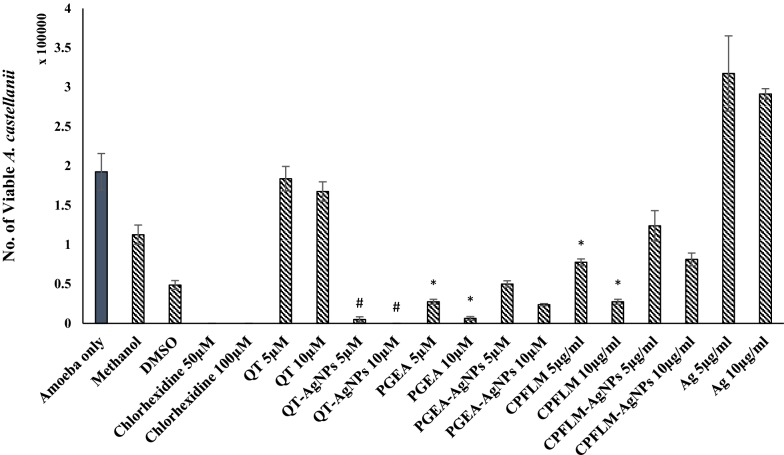
Effects of QT, PGEA, CPFLM, and the nanoconjugates of natural compounds mentioned above on the excystation inhibition of *A. castellanii* cysts. 1 × 10^5^ cysts were incubated with 5 and 10 μM (or mg/mL) of natural compounds or nanoconjugates for 72 h at 30 °C then the viability of *A. castellanii* trophozoites were determined by staining with 0.1% Trypan blue. The nanoconjugates of quercetin (10 μM) showed significantly increased results as compared to AgNPs and QT alone (#P < 0.05, using two samples T-test, two-tailed distribution). PGEA and CPFLM alone showed significant inhibition *of A. castellanii* excystation when compared to the negative control (cysts alone) (*P < 0.05, using two samples T-test, two-tailed distribution)

The compound QT alone did not exhibit a significant result in the excystation assay, however, 5 µM QT-AgNPs significantly reduced the number of viable amoeba cysts to 5 × 10^3^ as compared to the negative control (1.925 × 10^5^), AgNPs alone as well as QT alone (Fig. 6). Moreover, *A. castellanii* cysts were fully killed by treating with 10 µM of QT-AgNPs. On the other hand, PGEA and CPFLM decreased the viable number of amoeba cysts to 6.25 × 10^3^ and 2.75 × 10^4^ respectively, however their conjugation with AgNPs did not exhibit significant inhibition of excystation as compared to natural products alone (Fig. 6).

### Cytotoxicity of natural products and their nanoconjugates against human cells

**Fig. 7:**
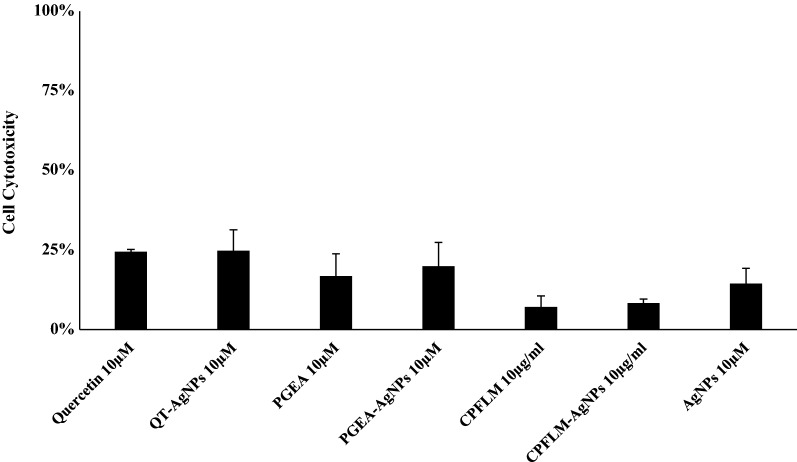
QT, PGEA, CPFLM and their silver nanoconjugates showed limited cytotoxicity to HaCaT cells. Briefly, 10 μM of test samples were added to HaCaT cells and incubated for 24 h at 37 °C in a 5% CO_2_ incubator. The compounds and nanoparticles showed less than 30% cytotoxicity at 10 μM

The compounds and their nanoconjugates while tested against human keratinocytes HaCaT cell line only showed minimal cytotoxicity as determined by LDH determination. None of the test samples were found to exhibit more than 30% damage to HaCaT cells (Fig. 7).

## Discussion

*Acanthamoeba* infections are challenging to treat mainly due to sturdy nature of cysts. The resistance of *Acanthamoeba* cysts has been a major obstacle in the antimicrobial chemotherapy. Several drugs have been developed to treat amoeba i.e. chlorhexidine, amphotericin B, pentamidine etc. (Niyyati et al. 2016), however, there are several difficulties faced by the treatment introduced including toxicity of the drugs towards human cells, limited penetration through the blood brain barrier, and the morphological transformation of the trophozoites into cysts which are more tough to kill (Khan 2006).

Silver nanoparticles have been recently known to enhance the antiacanthamoebic effects of the drugs (Anwar et al. 2018a). Using the help of the nanosized particles, the drugs for treatment may access to the target easier (Ahmed et al. 2016). Silver nanoparticles help to improve the ability of the compounds use for treatment as the drugs have poor bioavailability, therefore the conjugation with silver nanoparticles are likely to improve the transportation of the drugs to infected areas (Radwan et al. 2017). Recently, natural products coated nanoparticles have shown potent antiacanthamoebic effects. Tannic acid, Oleic acid, Cinnamic acid, Hesperidin, Naringin, Periglaucine A, and Betulinic acid coated nanoparticles have shown the step forward in the development of nanomedicine against challenging infections caused by *Acanthamoeba* (Padzik et al. 2018; Anwar et al. 2018b, 2019a, b; Mahboob et al. 2018). Moreover, various plant extracts coated nanoparticles have also shown promise in the drug discovery against *Acanthamoeba* infections. Nanoparticles coated with the extracts of following plants *Nigella sativa*, *Pterocaulon balansae*, *Jatropha curcas*, *Jatropha gossypifolia*, *Euphorbia milii*, *Aloe vera*, *Pongamia pinnata* have produced natural products based and safer alternatives against *Acanthamoeba* (Elkadery et al. 2019; Panatieri et al. 2017; Borase et al. 2013; Dinesh et al. 2015; Sundrarajan et al. 2015). However, the exact mechanism by which natural products coated nanoparticles exhibit antiacanthamoebic activities is yet not completely understood. Albeit, these in vitro studies suggest potential for drug development, but further mechanistic and in vivo studies are required before these can be recommended for any clinical implications.

The natural products used in this study QT, PGEA and CPFLM exhibited potent antiamoebic activities against *Acanthamoeba*. The killing effect of these compounds and extracts on amoebae hold promise as only low doses are required against both trophozoites and cyst stages. Moreover, these compounds and plants are widely distributed in a variety of environments which makes them easily accessible. According to the amoebicidal assays performed, QT, PGEA and CPFLM exhibited significant amoebicidal effects which were further improved in their nanoconjugates. The properties of the QT-AgNPs preventing the morphological transformation of trophozoites into cysts and vice versa have been found which is of immense importance in terms of drug development against *A. castellanii*. Notably, in excystation assay, 10 μM of QT-AgNPs had fully killed the trophozoites that transformed from the cysts in an advantageous environment (PYG; growth media). However, in the case of PGEA and CPFLM, the natural products alone showed better inhibition of encystation and excystation as compared to the nanoconjugates. QT and most of the flavonoids are known to possess antimicrobial activities against bacteria, fungi and parasites. Their mode of antiparasitic action against a common protist *Entamoeba histolytica* is considered to be a combination of various factors such as inhibition of energetic metabolism, cell proliferation and protein kinases. Moreover, several flavonoids are also known to damage DNA and induce apoptosis in *Entamoeba histolytica* (Martínez-Castillo et al. 2018). PGEA belongs to clerodane diterpene class of natural products. These compounds are known to produce permeabilization of fungal cell membrane (Cotoras et al. 2004). Although not much biological studies have been conducted on this compound, but it has also shown antifungal effects against phytopathogenic fungus *Botrytis cinerea* (Salah et al. 2003). CPFLM extract on the other hand is a known folklore medicine for eye inflammation and has been tested against various pathogenic bacteria and fungi. The high associated antimicrobial effects of CPFLM extracts are attributed to the presence of phenolic and polyphenolic compounds (Vivek et al. 2013).

In conclusion, results obtained in this study showed potential use of the natural compounds QT and PGEA, plant extract CPFLM and their nanoconjugates against *A. castellanii*. These natural products showed potent antiacanthamoebic effects as well as the inhibition of the encystation and excystation activity. Moreover, when conjugated with AgNPs, QT-AgNPs showed overall enhanced antiamoebic effects. However, mechanistic and in vivo studies should be carried out to determine the activity of the natural compounds and their nanoconjugates for drug development against *A. castellanii*.

## Data Availability

Data will be provided upon request on case to case basis.
